# A New Approach to the Computer Modeling of Amorphous Nanoporous Structures of Semiconducting and Metallic Materials: A Review

**DOI:** 10.3390/ma3010467

**Published:** 2010-01-15

**Authors:** Cristina Romero, Juan C. Noyola, Ulises Santiago, Renela M. Valladares, Alexander Valladares, Ariel A. Valladares

**Affiliations:** 1 Instituto de Investigaciones en Materiales, Universidad Nacional Autónoma de México, Ciudad Universitaria, Apartado Postal 70-360, México, D.F. 04510, Mexico; E-Mails: cromero22@gmail.com (C.R.); ulises_osa@yahoo.com.mx (U.S.); 2 Facultad de Ciencias, Universidad Nacional Autónoma de México / Ciudad Universitaria, Apartado Postal 70-542, México, D.F. 04510, Mexico; E-Mails: carlosnoyp@yahoo.com.mx (J.C.N.); renela6@yahoo.com (R.M.V.); avalladarm@unam.mx (A.V.)

**Keywords:** computational simulations, nanoporosity, porous semiconductors, porous metals, vibrational densities of states

## Abstract

We review our approach to the generation of nanoporous materials, both semiconducting and metallic, which leads to the existence of nanopores within the bulk structure. This method, which we have named as the *expanding lattice* method, is a novel *transferable* approach which consists first of constructing crystalline supercells with a large number of atoms and a density close to the real value and then lowering the density by increasing the volume. The resulting supercells are subjected to either *ab initio* or parameterized—Tersoff-based—molecular dynamics processes at various temperatures, all below the corresponding bulk melting points, followed by geometry relaxations. The resulting samples are essentially amorphous and display pores along some of the “crystallographic” directions without the need of incorporating *ad hoc* semiconducting atomic structural elements such as graphene-like sheets and/or chain-like patterns (*reconstructive* simulations) or of reproducing the experimental processes (*mimetic* simulations). We report radial (pair) distribution functions, nanoporous structures of C and Si, and some computational predictions for their vibrational density of states. We present numerical estimates and discuss possible applications of semiconducting materials for hydrogen storage in potential fuel tanks. Nanopore structures for metallic elements like Al and Au also obtained through the *expanding lattice* method are reported.

## 1. Simulating Nanoporous Materials

The understanding of complex systems like porous and amorphous materials has been furthered more by experimental work than by theoretical efforts. The experimental drive has led to the generation of a multitude of disordered materials whose structures are at best determined at the global level with the use of techniques like the scattering of electrons, neutrons, *etc*. through their radial, or pair, distribution functions (RDFs). Clearly, such functions do not represent a unique structure, since many atomic topologies lead to the same or, at least, indistinguishable RDFs. So how can a specific disordered atomic structure be determined experimentally? Presently, there is no way. It is here where theoretical methods can be extremely useful. Until very recently it was practically impossible to imagine the generation of an atomic topology for an amorphous or a porous material; attempts were based on approximations that were questionable and consequently unreliable. With the advent of sophisticated computer codes and powerful computers an otherwise unknown insight was gained; however, approximations are still used that essentially limit the findings to the nanoregime. As codes improve and computer power increases it will become possible to study the meso and the macro regimes with the consequent benefit for the dialectic relation between theory and experiment.

From the essential view point one needs to know the structure of the material before one can attempt to calculate its properties. Even then the multiplicity of structures that have indistinguishable RDFs forces one to calculate several properties with the hope of finding results that agree with experiment, and thereby being able to conclude that the corresponding atomic topology is the adequate one to describe the material. In what follows we present some efforts that our group has made to generate porous materials in the nanoregime that lead to amorphous porous materials, both semiconducting and metallic, that manifest features similar to experiment. Some properties have been calculated to compare with experiment and the results seem promising. Since our approach is *ab initio* the computational demand is onerous but it removes the necessity of adjusting parameters and potential energy expressions which sometimes mask the true nature of the subject and makes the results and the approach non transferable. We use the supercell approach and its size limits the size of the features that we can describe. Moreover, this approach introduces a spurious periodicity that should be dealt with usually by ignoring it and by rejecting any results it influenced. We optimistically look forward to the day when the *ab initio* codes can efficiently handle many more atoms, the computers have considerably more power, the supercells used are much larger and the simulations finally describe the real amorphous or porous material.

In a very recent issue of the *Materials Research Society* (MRS) *Bulletin* [1] a selection of papers on hard materials with tunable porosity was published and a review of the present situation of some technologically relevant areas was given. Among these areas catalytic and optical applications were considered, formation of nanoporous metals by alloy corrosion was reviewed, methods of formation of meso- and macroporous ceramics was presented, and most important for our present work, modeling methods of amorphous porous materials was dealt with. To avoid unnecessary repetitions we refer the technologically minded reader to that issue. For the computationally minded reader we can say that in the paper on modeling materials contained in this journal no *ab initio* methods are cited, which shows the difficulty of incorporating these concepts into the computer modeling of amorphous and porous materials. Most proposals reported in the literature are what we are going to call descriptive, since they use the experimental results to then construct an approach to describe (find) the structure designed in the laboratory; therefore these methods need the experimental input and are preferentially based on the Monte Carlo method. Two widespread approaches are mentioned frequently in the literature: one is the so called *reconstructive* simulations method; the other is the *mimetic* simulations approach. Our method is neither, so we call it the *transferable* approach since in principle we claim it to be useful for diverse materials and several formation processes rather than for a specific one; in this sense our method is more predictive than descriptive and it is herein that its advantage resides; however, the limitations are clear. Being *ab initio* we cannot handle too large a sample and therefore the supercells used (the features studied) usually involve no more that 500 atoms if a non self-consistent method is utilized. Another limitation that should be kept in mind is that up to now we can generate porous structures that are amorphous but polycrystalline porous samples are not yet covered by our approach. Finally, there are two bulk quantities that can be varied and their consequences studied: the temperature of the molecular dynamics (MD) process and the percentage of porosity of the material.

We started to study nanoporous structures of carbon to investigate their possible use as a storage “fuel tank” for hydrogen [2,3]. It is well known that activated carbon has been used for a long time as a reactive cleaning agent to get rid of unwanted by-products in catalytic processes. This reactive behavior makes the study of porous carbon as a fuel tank to store hydrogen or to trap other contaminating substances a technologically important subject. Around the time we began our investigations an experimental work was published concerning hydrogen adsorption in different carbon structures [4] making the subject even more appealing since a comparison of our results with experiment could then be made possible [3]. Due to its chemical valence carbon appears in a variety of atomic structures and the multiplicity of bond types leads to amorphous carbon, nanotubes, nanoporous carbon and graphene nanostructures, to mention a few. One would expect that these structures would manifest themselves as amorphous nanoporous carbon since the atomic structure is related to the density, and this varies notably depending on the porosity of the samples.

Porous silicon may be an alternative in the quest to find an adequate fuel tank for hydrogen. Its amorphous porous state (ap-Si) has also been the subject of our interest, since experimentally it is well known [5], and theoretically not as well studied. We have computationally generated samples of ap-Si with 50% porosity from crystalline supercells that were carved along several crystallographic directions. It was found that the electronic gap increased when they were first relaxed, then amorphized, and finally relaxed again [6]. However, due to the way in which our nanoporous samples were constructed the preferential initial carving predetermined the orientation of the pore and that is why we were forced to think differently to avoid this limitation.

The simulation of a material like porous Si, which in itself is not uniquely structured or clearly characterized [7], poses a very difficult but interesting challenge. Experimentalists claim that porous silicon may be crystalline or amorphous, but the degree of crystallinity (amorphicity) is difficult to establish. Some even claim that samples with 90% porosity are crystalline, which makes the role of the bulky backbone essential and forces any simulation to contain many atoms to describe it adequately.

Meanwhile the experimental quest for new crystalline materials with a variety of porous structures has continued and has acquired an important dynamics, mainly due to their potential usefulness in various applications that go from catalytic processes to storing and filtering atoms and molecules. In a recent paper the structure and catalytic activity of a highly porous silicogermanate was reported [8]. Corma and coworkers have synthesized a silicogermanate zeolite identified as ITQ-33 that could be a molecular sieve with 18- and 10-member rings, which may have a relevant role in the production of more diesel and less gasoline during catalytic cracking of vacuum gas oil (VGO). The zeolite obtained is very small, of the order of micrometers, and this could pose an obstacle for some applications of this material. Since the synthesis is recent this may be an indication of the difficulties encountered in the experimental efforts to produce highly porous crystals or crystallites.

In view of this our main concern has been to devise a process to generate nanoporous structures of covalent materials whose final atomic topologies are independent of the orientation of any initial carving described abundantly in the literature. Actually, carving along some definite crystallographic direction in otherwise crystalline samples is a widespread method to computationally generate porous materials but predisposes the resulting structure to the initial manipulation. We have found a computational process that leads to structures with different porosities (different densities) and that show pores along some of the original crystallographic directions of the supercell; these pores appear in a natural way since there is no initial carving of pores in the material. We identify this process as the *expanding lattice* approach which is presented in the next section.

The question is: what is the general validity of the expanding lattice approach? We had only tested it for silicon and carbon, using both *ab initio* techniques and parameterized classical Tersoff potentials, but we needed to test it in other covalent and non-covalent materials. Thus we decided to apply the expanding lattice method to metallic systems in the nanoporous regime. These materials are being investigated for their potential usefulness as reactive agents in the biophysical world due to their chemical reactivity in nanocluster form. Over and above their potential applications these metallic nanoporous systems have interesting characteristics which make them appealing in simulational efforts such as the effect that the porosity has on their electronic, optical and vibrational properties.

Historically, porosity in metallic systems was considered undesirable since it is associated with corrosion problems [9,10,11]. At present porosity is not only desirable, but is the subject of studies to find methods to produce porous metallic structures with a well controlled pore size, and sometimes with a well controlled pore form [12,13,14,15]. Most recent publications use the dealloying technique to generate metallic nanoporous systems [14,16].

Between 2001 and 2004 Erlebacher *et al.* [17,18] proposed a continuum model that led to the prediction of atomic topologies consistent with experiment and with theoretical simulations of alloy dissolution. They showed that nanoporosity in metals has to do with an intrinsic dynamical pattern, the spinodal decomposition. Erlebacher and coworkers reached these conclusions by applying their model to Ag-Au alloys using Monte Carlo simulations.

In 2007 Crowson *et al.* [19] set out to answer the question: if we start with an Ag-Au alloy with a certain cell length and remove all the Ag atoms, what would the resulting cell length be for the remaining Au atoms? They carried out MD based on interatomic potentials of the embedded atom kind that allowed them to handle thousands of atoms [20]. In 2009 they applied this technique to determine the mechanical stability of nanoporous metals as a function of the size of the ligaments, or backbones as they are also known in other areas [21]. To the best of our knowledge these are all the computational efforts applied to nanoporous metallic systems.

## 2. The *Expanding Lattice* Approach [23]

The expanding lattice approach had its origin in the extensive studies of amorphous materials that our group has carried out [22]. In the process we noticed that the lower the density of the generated amorphous materials the more porous they were, so we decided to systematize our procedure by expanding the lattice along the three directions in such a manner as to generate a supercell with the porosity (density) desired. Assume we start out with a cubic supercell of edge length *a*; if we now expand each of the three sides by a factor *l^1/3^*, correspondingly increasing the interatomic distances, then the final volume will become *a^3^l*. If *l* is larger than 1 (expansion) the final density will be smaller that the initial one: *ρ_f_ = ρ_i_/l*. Once this new supercell is constructed an MD process is started at a constant temperature dictated by the melting temperature of the material in bulk and by the desired nature of the final structure. At the end of the MD process a porous supercell is generated where the atoms rearrange themselves so as to minimize their energy at the temperature at which the MD was carried out. To ensure that we generated a minimum energy structure we perform a geometry optimization of the sample. This is the simple essence of our novel approach [23].

From the bulk viewpoint, expanding a solid implies the application of negative pressures. However, in our case rather than applying a negative pressure to the material we simply create an unstable structure by increasing the volume (and increasing the interatomic distances) with a larger energy than the original crystalline structure and through MD we foster the rearrangement of the atoms towards a structure with low energy, congruent with the temperature of the MD process. Once the volume is increased we maintain this value fixed and the excess energy is liberated through the dynamics process. Finally a state is reached that can be described as a minimum energy structure by looking at the evolution of the energies in the system as a function of the number of computational steps.

The *ab initio* MD is performed using **FastStructure** [24], a density functional code based on the Harris functional that allows simulated annealing/molecular dynamics studies with quantum force calculations. Harris’ molecular dynamics code employs linear combinations of atomic orbitals (LCAO) as basis functions, and allows the random heating, annealing and quenching of periodic supercells. The LDA parameterization of Vosko, Wilk and Nussair has been used in all our simulations [25]. In general when the core is considered relaxed only the deep core electrons are regarded as frozen; when an all electron calculation is carried out then all the electrons are included in the calculation and this can be done for low atomic number elements, like carbon. Additional parameters like the value of the time step, the molecular basis set functions, cutoff radius, pseudopotentials or core potentials, *etc*, were adjusted according to the specific material under study and will be discussed specifically for each material.

In this work we review the expanding lattice approach applied to semiconductors, like carbon and silicon and also to pure metals, like aluminum and gold. Our original approach for semiconductors involved *ab initio,* quantum mechanically based MD which restricted us to periodic supercells of some hundred atoms that can be handled with present ordinary computational resources. Thus any property whose study requires a very large number of atoms may not be representatively calculated from our structures. However, since we wanted to see the relevance of the size of the supercell we decided to do some expanding lattice calculations using a well proven and well accepted parameterized classical interatomic potential, the Tersoff potential, designed primarily for Si and C.

Our results indicate that both quantum mechanically and classically the pores in carbon and silicon are freely formed preferentially along certain “crystallographic” directions. The RDFs are reported; for carbon they give information concerning the nature of the bonds, since the structure of the first peak indicates the presence of double and triple bonds in addition to the resonant graphite-like bond; for silicon the structure of the first peak of the RDF indicates different bond lengths on the pore surface and within the bulk of the sample. Furthermore the highly localized nature of the first peaks suggests the existence of a crystalline-like structure (crystal-like short range order) in the porous samples, whereas the broad nature of the second peaks indicates the amorphous nature of the samples.

For pure metals we have recently found several fascinating features. First of all when we generate a 50% porosity 108-atom aluminum supercell at a temperature of 918 K we find a porous structure that resembles layers, perhaps reminiscent of the aluminum foam structures. Gold on the other hand does not show an emphatic through-pore structure, but the results may be associated to the presence of dendritic pores. Work is in progress in order to understand and discern these atomic structures.

As mentioned, the advantage of our method is that instead of describing porous materials, as with *reconstructive* or *mimetic* simulations, we attempt to predict their structures as a function of the MD temperature, or the porosity, regardless of the type of the atomic constituents. That is why we describe our approach as *transferable*.

## 3. Semiconducting Materials

In dealing with the *ab initio* simulation of porous carbon and silicon, the following procedure was carried out. We first formed a crystalline diamond-like supercell, with periodic boundary conditions, 216 atoms, *a* = 13.48 Å for C and *a* = 20.53 Å for Si. Then the volume of the supercell was doubled by lengthening the cell edge *a* to 2^1/3^*a* and the interatomic distances increased accordingly. Since the structure obtained in this manner (half the original density or 50% porosity) is metastable we performed *ab initio* MD at 300 K for silicon and at 1,000 K for carbon during 100 steps at 4.0 fs per time step. Then the atomic structures were allowed to relax into minimum energy structures and pores appeared along well defined crystallographic orientations with respect to the original crystalline supercell.

Since most of the experimental results show pores in *n-* or *p-*doped silicon our results for the pure materials have no clear experimental counterpart so far and therefore should be considered predictive. The RDFs obtained reveal amorphous porous structures with highly localized first peaks. The parameters used in the MD runs are minimal basis functions for Si and standard basis functions for C, sets used in our previous work to reproduce the experimentally reported positions of the first peaks in amorphous materials [26]. Also, a full core for each element, a cutoff radius of 3 Å for carbon and 5 Å for silicon and the Harris functional with a linear combination of atomic orbitals in a non selfconsistent approach were utilized during the runs [24].

When trying to disorder the carbon supercell we observed that the MD process carried out at 300 K practically left the structure unaltered, so we increased the MD temperature to 1,000 K. The rule of thumb is that the minimum constant temperature at which the MD should be carried out to foster the atomic rearrangement is no less than 20 to 25% of the melting temperature.

### 3.1. Nanoporous Carbon

Within the wide variety of structural forms of carbon we find the so-called activated or nanoporous carbon. The importance of studying these materials lies in the fact that they have a number of applications such as filters, catalysts, bioreactors, cells, electrodes, surgical implants, *etc*. [27], due to their unique properties, their large surface area being one of the most outstanding. The most common way to prepare nanoporous carbon is through the pyrolysis of an appropriate precursor such as polyfurfuryl alcohol, sucrose or polyvinyldichloride. The selection of this precursor and the pyrolysis temperatures are the main factors responsible for the microporous structure of these materials [28]. Petkov *et al*. [29] reported nanoporous structures of carbon obtained from neutron diffraction data and produced by pyrolysis of polyfurfuryl alcohol at three different temperatures (400, 800 and 1,200 °C). They found that carbons processed at 400 °C have a heavily distorted non-planar structure while the carbons produced at 800 and 1,200 °C consist of stacked, more or less curved, graphene sheets. It would seem reasonable then that in order to simulate experimentally designed nanoporous carbon more than one approach should be considered.

In what follows we first present the results of the simulation using the *ab initio* expanding lattice approach of a 50% porosity carbon sample as reported in ref. [23]. Next we report simulations for other values of the porosity guided by the experimental data of Petkov *et al.* [29] to discern what parameters are important in the simulations presented here.

#### 3.1.1. A 50% Porosity Carbon Sample [23]

**Figure 1:**
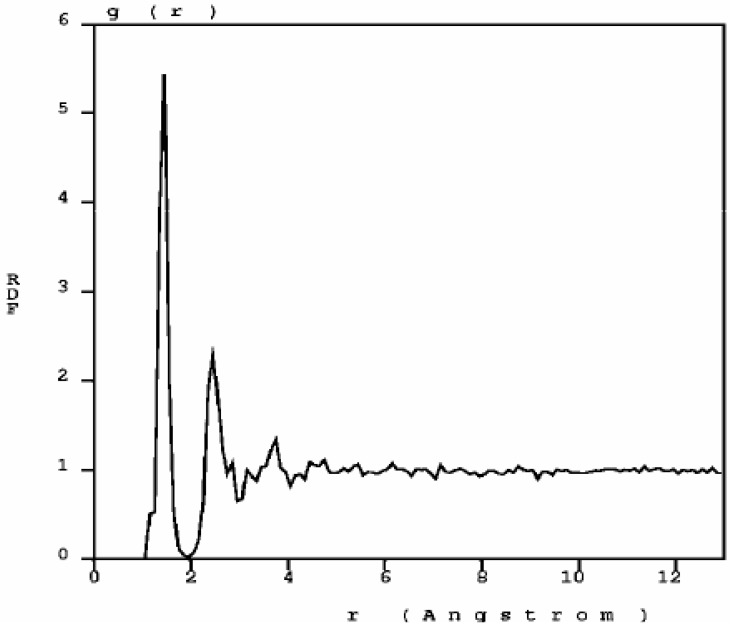
The atomic topology (RDF) for nanoporous carbon is more typical of an *amorphous* (porous) sample. There are three well defined peaks, and a minimum between the first and the second peaks as in typical amorphous materials [23].

**Figure 2:**
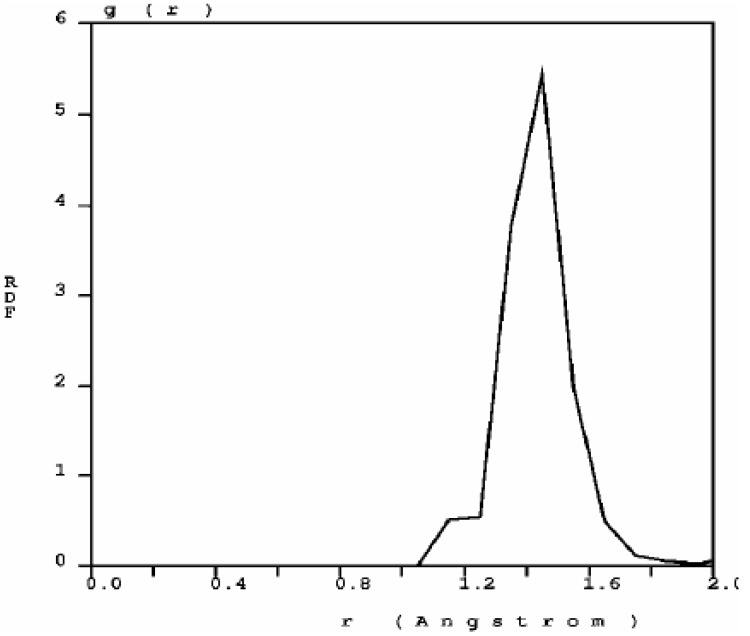
Bonding details of the first peak of the RDF of nanocarbon. There is a structure in the first peak that may be associated to graphitic bonds (1.45 Å), double bonds (1.35 Å) and triple bonds (1.15 Å). The diamond interatomic distance is 1.54 Å.

The RDF of nanoporous carbon obtained by the expanding lattice method (Figure 1) has three prominent peaks and a well defined minimum between the first and the second peak; the distance at the minimum is the value used to define a bond between nearest neighbors. When one looks at the first peak of the carbon RDF in more detail (Figure 2) the following features appear. There are three peaks that may be associated to three different bonding states: single bonds (1.45 Å), contracted with respect to the crystalline diamond-like value of 1.54 Å, but close to the interatomic distance in graphite, 1.41 Å; double bonds at 1.35 Å, to be compared with 1.34 Å for C=C; triple bonds at 1.15 Å, to be compared with 1.20 Å for C≡C [30]. The number of nearest neighbors with no multiple bonds, as determined by the corresponding height at *r* = 1.45 Å, is larger than the rest, which indicates that they may be located mainly within the bulk forming graphitic rings, whereas the atoms with double bonds and triple bonds are mainly located on the surfaces of the pores since the pores are small compared to the backbone.

**Figure 3:**
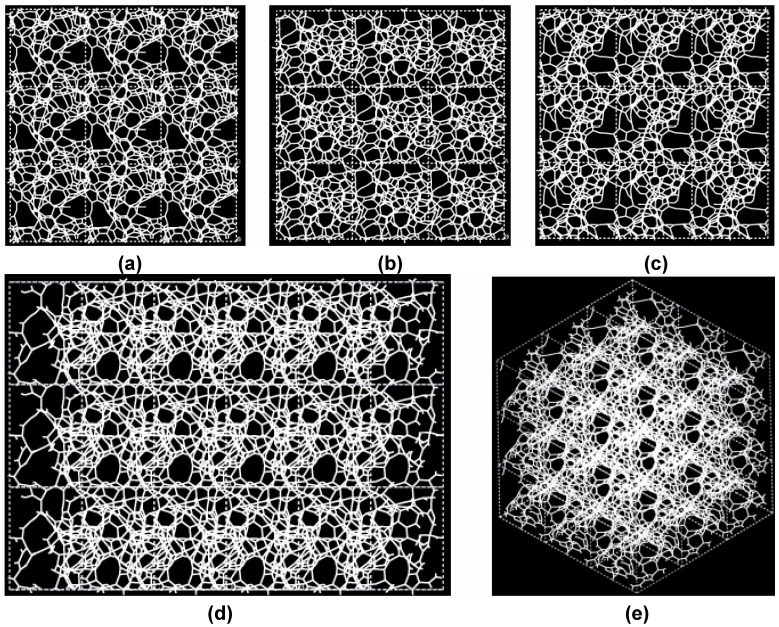
Pore structure obtained for 3 × 3 × 3 carbon cells. There are through pores in the (a) x and (c) z directions and smaller pores along the (b) y, (d) zy and (e) xyz directions.

If one looks at the atomic structure of nanoporous carbon depicted in Figure 3, an obvious conclusion is reached: the number of 6- and larger-atom rings, is high; this should not be a surprise since the density of these samples is half the density of diamond and therefore closer to the density of graphite whose interatomic distance in the plane is 1.41 Å.

In Figure 3 the tubular through pore structure of this simulated nanoporous carbon is presented along certain crystallographic directions referred to the original primitive crystalline supercell. Clearly, dendritic pores are difficult to observe in these representations, since they do not appear along specific directions. Pores in these structures are not as well defined as for silicon (see next section) but nevertheless they exist. It seems that despite the contraction of the nearest neighbor bonds in nanoporous carbon (Figure 2) low density carbon tries to avoid the voids (pores) by expanding and filling, as much as possible, the available space. For silicon the opposite seems to occur, assuming that dendritic pores play similar roles in both materials. The pores are more noticeable in the (a) x and (c) z directions and smaller ones appear along the (b) y, (d) zy and (e) xyz directions. Nevertheless this tendency to have through pores along certain preferential directions can be observed.

#### 3.1.2. Simulations Led by Experimental Work [31]

The versatility of the final structures as a function of the processing temperatures obtained by Petkov *et al.* [29] and mentioned above (Section 3.1), indicates that these structures may not be in a minimum energy state, but rather more likely they are in metastable arrangements. This is the typical situation for *reconstructive* simulations where the addition of *ad hoc* elements such as chain-like or layer-like atomic arrangements, combined with Reverse Monte Carlo methods, lead to a final topology in agreement with experiment. Some simulational studies employ Monte Carlo (MC) and Reverse Monte Carlo (RMC) techniques using chains of amorphous polymers [32] and graphene sheets [33].

For this simulation the expanding lattice approach was applied to a crystalline diamond supercell with 216 atoms and periodic boundary conditions whose volume was increased by lengthening the cell edge from *a* = 10.67 Å to (2.569)^1/3^*a* = 14.62 Å to obtain the desired density of 1.38 g/cm^3^. This represents a porosity of 61.1% of the original sample and corresponds to the experimental data. We performed *ab initio* MD at 1,000 K during 295 steps with a time step of 4 fs. When the simulation was complete we optimized the structures by energy minimization. We used a full electron core to carry out an all electron calculation, a cutoff radius of 3 Ǻ and standard basis set functions.

**Figure 4:**
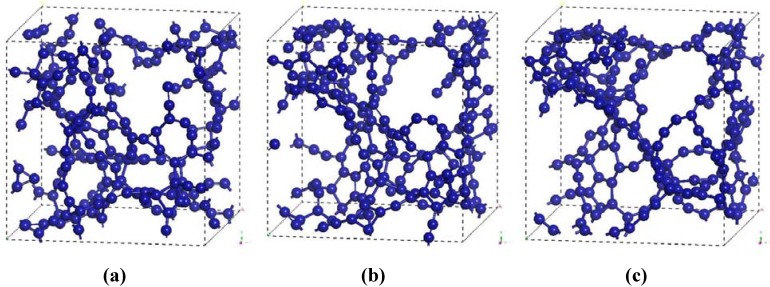
Evolution of the atomic structure of carbon with 61.1% porosity as a function of the number of simulation steps. Optimized structures are shown for (a) 100, (b) 200 and (c) 295 simulation steps.

**Figure 5:**
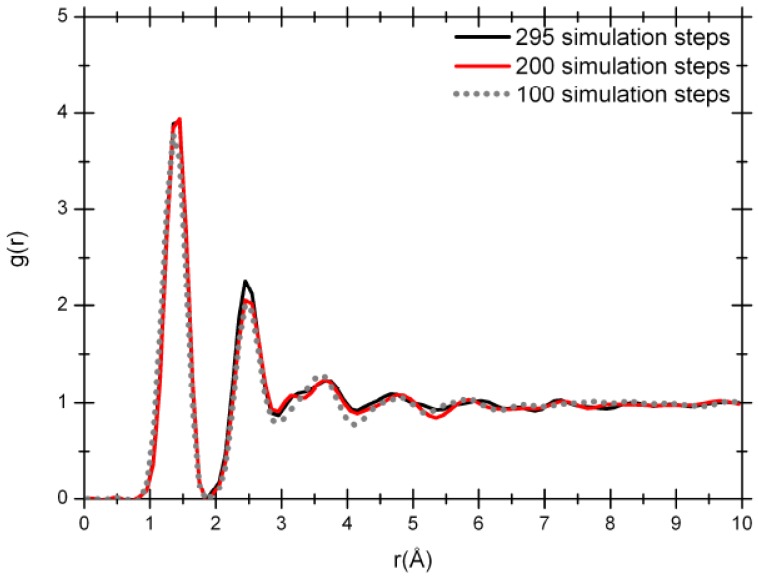
RDFs for 61.1% porosity nanoporous carbon with a density of 1.38 g/cm^3^. The dotted line is the RDF for 100 simulation steps; the dark gray solid line (red for the online version) is the RDF for 200 simulation steps and the black solid line is the RDF for 295 simulation steps.

The atomic topology of the porous structure was followed during the MD run to see the effect of the number of steps on the topological evolution of the supercell. After geometry optimization at 100, 200 and 295 simulation steps we obtained the pictorial view of Figure 4 that shows how the system evolves. The Fourier-smoothed RDFs for each of these three simulation steps are shown in Figure 5. We can see that there are three well defined peaks in the RDFs. The positions of the maxima of the first, second and third peaks for the optimized structure at the end of 100 simulation steps are 1.35/1.45, 2.45 and 3.65 Å, while the two structures for the 200 and 295 simulation steps give the same values of 1.45/1.35, 2.45 and 3.65 Å. A fourth peak can be identified, but beyond 8 Å the RDFs are essentially structureless.

It should be mentioned that the RDFs for the three simulation stages before Fourier smoothing also have first-neighbor peaks with well defined structures as for the 50% porosity sample. For all three of them three distinct peaks can be observed: one at 1.15 Å, another at 1.35 Å and finally a higher one at 1.45 Å. As before, an analysis of the structures to identify the origin of these three peaks seems to indicate that the short distance peak may be associated to triple-bonded carbons, the intermediate distance may be due to double-bonded carbons, and the peak located at 1.45 Å may be due to carbons that are part of graphite-like layered structures.

The radial distribution functions at 100, 200 and 295 simulation steps show a topology typical of amorphous carbon with the short range order mentioned above. The shape of the RDFs for 200 and 295 steps is practically the same but there is a pronounced difference between the position of the first peak for the 100 simulation-step RDF and the 200 or 295 simulation-step RDFs. That difference could be attributed to the proportion of carbon atoms linearly and trigonally coordinated. The amount of linearly coordinated carbon atoms is significant in the 100-step simulated sample in contrast to the 295-step sample: 40.74 *versus* 29.62%, respectively, indicating the presence of carbon chains in the 100 step sample. The percentage of carbon atoms trigonally coordinated in the two samples is 51.85 *versus* 61.11%, respectively. These results are connected to the coordination number ─ the area under the first peak of the radial distribution function *J(r)* = 4πr^2^*g(r)* ─ which is 2.66 for the 100 simulation-step structure and 2.78 for the 295 simulation-step structure; this last value is closer to the coordination number of the crystalline structure of graphite.

**Figure 6:**
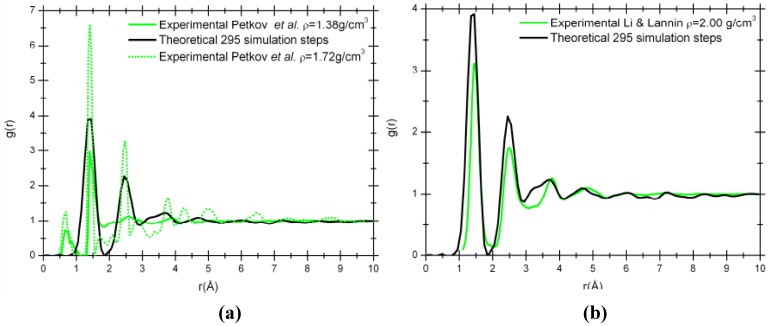
Comparison of our 295 step simulated RDF, g(r), with a density of 1.38 g/cm^3^ (black solid line) and the experimental RDFs. (a) Experimental nanoporous carbon with a density of 1.38 g/cm^3^ (green solid line) and 1.72 g/cm^3^ (green dotted line). (b) Experimental amorphous carbon with a density of 2.00 g/cm^3^ (green solid line).

Figure 6 compare our simulation results to the experimental RDFs from Petkov *et al*. [29] for two densities, 1.38 g/cm^3^ and 1.72 g/cm^3^, obtained from the pyrolysis of polyfurfuryl alcohol at 673 K and 1,073 K, respectively [Figure 6 (a)]; and to the experimental RDF from Li and Lannin [34] for a density of 2.00 g/cm^3^ obtained by neutron diffraction on amorphous carbon films [Figure 6 (b)]. Table 1 compares the parameters associated with the densities, the first peak positions, the coordination numbers and the percentage of carbons trigonally coordinated, presumably belonging to carbon sheets. The proportion of atoms trigonally coordinated obtained in our simulation is closer to the values for the experimental sample with a density of 1.38 g/cm^3^, while our coordination number compares favorably with the experimental sample of 1.72 g/cm^3^. On the other hand, the simulated RDF matches the amorphous experimental sample of Li and Lannin [34] better than the porous experimental RDFs obtained by Petkov *et al.* [29]. We are presently exploring the role of the (constant) temperature at which the MD is performed to see if a more “crystalline” porous structure can be obtained.

**Table 1:** Comparison of various parameters associated with densities, first peak positions,coordination numbers and percentage of carbon atoms trigonally coordinated.

	This work	Petkov *et al.* [29]	Petkov *et al.* [29]	Li and Lannin [34]
Density (g/cm^3^)	1.38	1.38	1.72	2.00
First peak position (Å)	1.45/1.35	1.41	1.41	1.46
Coordination number	2.78	1.9 ± 0.2	2.6 ± 0.15	3.34
Atoms trigonally coordinated (%)	61	63	86	—

**Figure 7:**
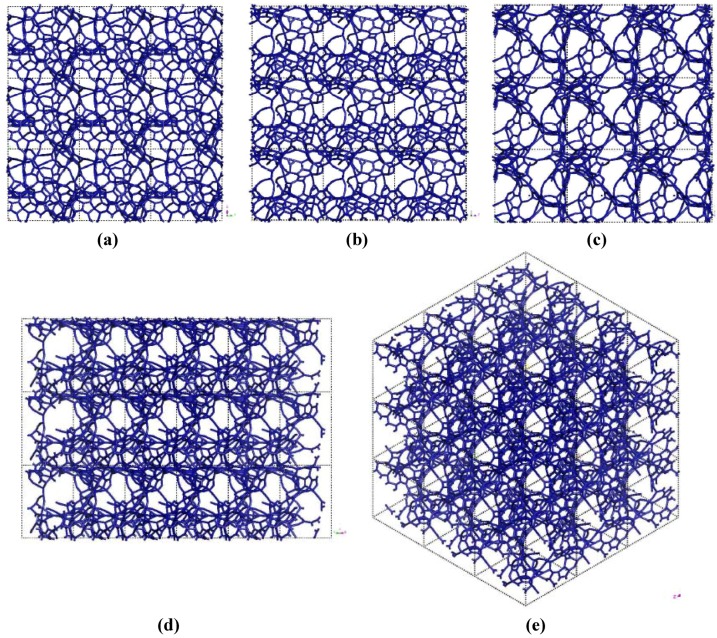
Pore structure obtained for 3 × 3 × 3 low density carbon cells. Prominent through pores exist along (c) z, (d) yz and (e) xyz, and smaller ones along (a) x and (b) y directions.

A tubular open pore structure still prevails along certain crystallographic directions with respect to the original primitive supercell. Figure 7 shows that pores are prominent along the (c) z, (d) yz and (e) xyz directions and smaller pores appear along (a) x and (b) y directions for the 295 cell.

### 3.2. Nanoporous Silicon

In what follows we first present the results of the *ab initio* simulation of a 50% porosity silicon sample using the expanding lattice approach as reported in Ref. [23] where more detailed information can be found. Next we report simulations carried out with the Tersoff parameterized interatomic potential also for a 50% porous sample to compare with the first principles simulation.

#### 3.2.1. *Ab Initio* Simulations [23]

**Figure 8:**
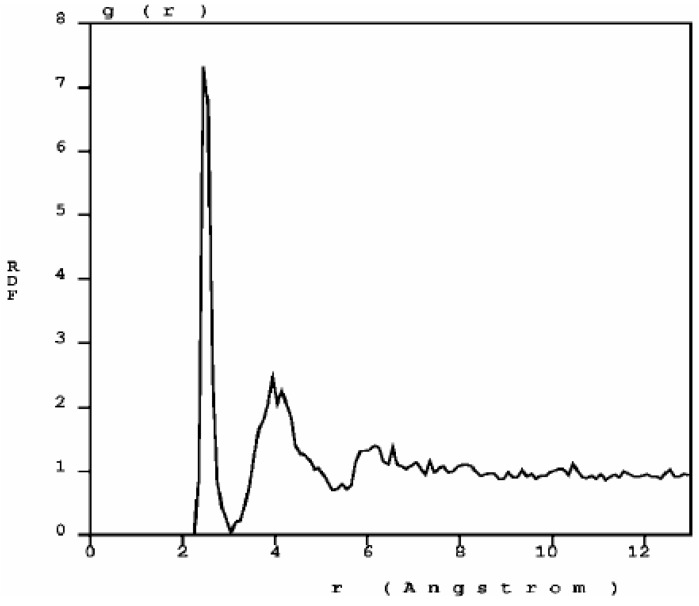
RDF for nanoporous silicon. The atomic topology is more typical of an amorphous porous sample since, although the first peak indicates highly localized nearest neighbors, (crystalline-like behavior), the second peak is broader, like those found in amorphous materials, especially in Si [26].

The topological results for the expanding lattice *ab initio* approach for silicon are depicted in Figure 8 to Figure 10, where the RDF and the fine structure of its first peak are presented, together with the pores formed with the expanding lattice approach.

**Figure 9:**
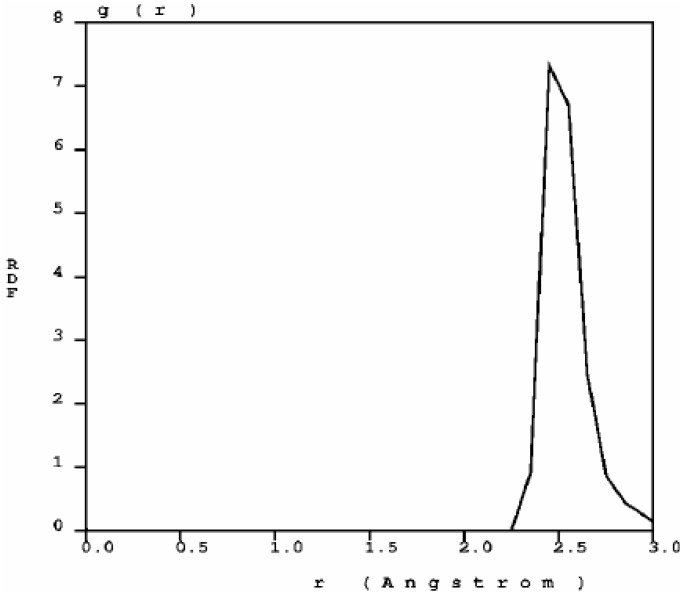
The first peak of the RDF of nanosilicon has a structure due to the interatomic distances on the surface of the pore (2.55 Å) and within the bulk (2.45 Å). It has been shown that the larger the number of atoms in the supercell, the interatomic distance becomes closer to 2.35 Å, the crystalline value [35].

Figure 8 shows the RDF for nanoporous silicon. Figure 9 presents the fine structure of the first peak of the RDF where the interatomic distances on the surfaces of the pores, 2.55 Å, and within the bulk, 2.45 Å can be identified and are larger than the crystalline diamond-like value of 2.35 Å. This identification was done by obtaining the RDFs for pore surface atoms and for bulk atoms (E.R.L. Loustau, private communication). More recent studies have shown that the larger the number of atoms in the cell (lower porosity), the interatomic distance becomes closer to the crystalline value. This is an example of the obvious fact that the size of the backbone plays an important role in determining the structure of nanoporous silicon [35].

**Figure 10:**
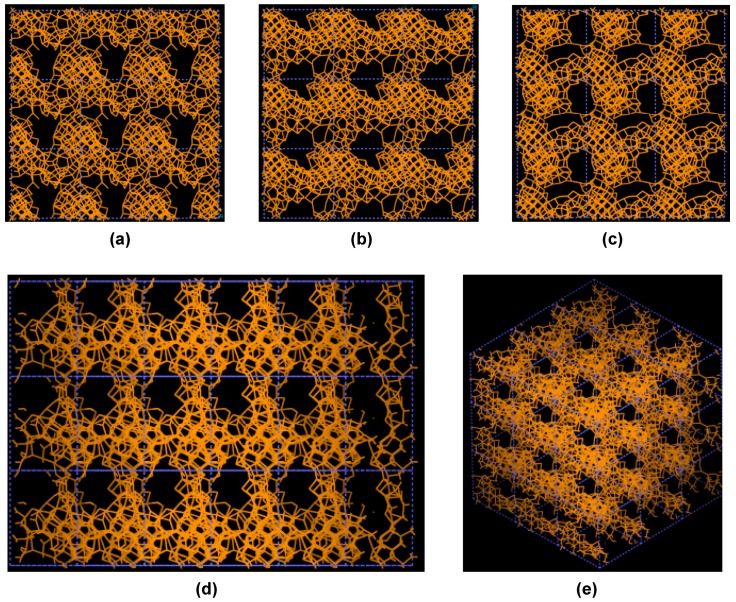
Pore structure obtained for 3 × 3 × 3 silicon cells. The through pores here are found in the (a) x, (b) y and (c) z directions as well as in the (d) yz and (e) xyz directions.

Figure 10 shows the tubular pore structure of nanoporous silicon for certain crystallographic directions referred to the original primitive supercell. As for carbon, dendritic pores are difficult to observe, since they do not appear along well defined directions. Pores in these structures are much more noticeable than in carbon. They are more prominent along the (a) x, (b) y and (c) z directions and also along the (d) zy direction; smaller pores appear along the (e) xyz direction. As mentioned before, despite the expansion of the nearest neighbor bonds in nanoporous silicon (Figure 9) it seems that low density silicon does not avoid the voids whereas for carbon the opposite seems to occur, assuming that dendritic pores play similar roles in both materials. Since the volume of a silicon atom is larger than that of a carbon atom, the total volume of 50% silicon atoms is larger than the volume of 50% carbon atoms. This may also contribute to the impression that the pores in silicon are larger than in carbon.

#### 3.2.2. Tersoff-Based MD Simulation [36]

Efforts to model p-Si have been carried out using quantum methods [6,23,37], as well as classical methods [38,39,40,41,42,43]. In the present work, in addition to the *ab initio* method described above [23] we decided to use an interatomic classical, parameterized potential developed by Tersoff [44,45] to describe a-Si and apply it to the generation of p-Si using a classical MD dynamics process acting on a supercell of 1,000 atoms with periodic boundary conditions. Several studies show that the Tersoff potential is applicable to the generation of non crystalline structures of silicon such as silicon atomic clusters [43] and amorphous silicon [44,45], but as far as we know the first application to p-Si is the one in Ref. 36. The use of empirical potentials allows simulating the interaction of thousands of atoms, which is useful if one wants to study large pores and properties such as vibrational frequencies; the Tersoff potential has been proven useful in the study of vibrational properties of a-Si [46,47,48,49,50].

As before, to generate the 50% porous sample we start out with a crystalline supercell with a density of 2.33 g/cm^3^ and 1,000 atoms with periodic boundary conditions. The cell volume is doubled multiplying the original edge by a factor of 2^1/3^, the number of atoms is held fixed and the interatomic distances are increased by the same factor in such a way that the fractional coordinates remain unaltered. The density of the new supercell, 50% porosity, is then half the value of the original density.

In the resulting structure the atoms are in non-equilibrium positions. Then the supercell is subjected to classical MD processes with N and V held constant and at a constant temperature of 300 K. In this case the interaction between atoms is modeled by the Tersoff potential contained in the OXON package [51]. The parameters used for the potential are those reported in Ref. [45]. The atomic velocities are initiated randomly and then fitted to a Gaussian velocity distribution at the corresponding temperature. The force on a particular atom is the negative value of the gradient of the potential. The equations are integrated using the Verlet algorithm [52] with a time step of 8 fs. Subjected to this process the atoms were displaced to positions that minimized the total energy of the system and the kinetic energy values converged to a value such that the initial structure evolved into an equilibrium configuration. In order to obtain the minimum energy configuration the structure was relaxed using the conjugate gradient method; the maximum allowed force on any specific atom was 0.01 eV/Å.

**Figure 11:**
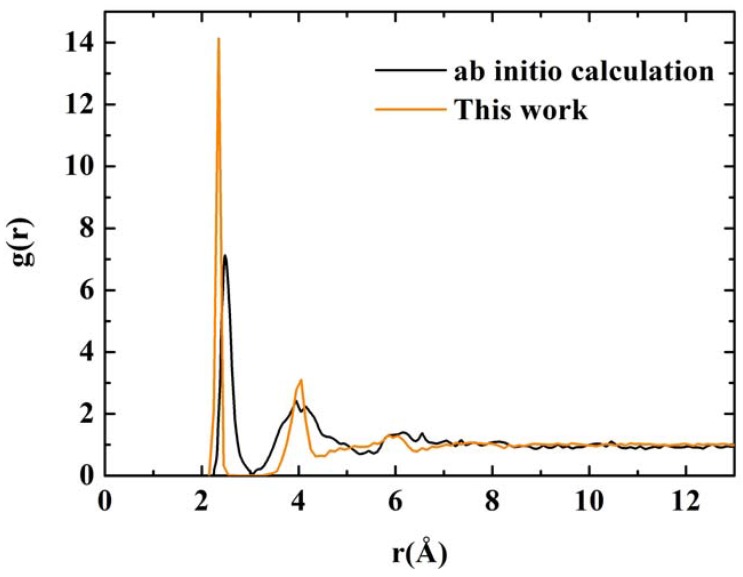
RDFs for nanoporous silicon. The black line is the RDF obtained by Valladares *et al*. [23] by means of an *ab initio* calculation on a 216 atom supercell (see Figure 8). The orange line was obtained using the Tersoff interatomic potential and a 1,000 atom periodic supercell.

Once the porous structure was obtained, the structural properties were determined first, and then the vibrational properties. In Figure 11 we show two RDFs for porous silicon, one calculated with *ab initio* techniques by Valladares *et al.* [23] on a 216 atom supercell and the other using Tersoff on a 1,000 atom supercell. In contrast to the quantum calculation the Tersoff RDF shows that the first and second neighbors (short-to-middle range) are much more localized. This can be seen by the sharpness of the peaks whose maxima are at 2.35 Å and 4.05 Å (compare these values to the crystalline distances between nearest neighbors, 2.35 Å; second neighbors, 3.84 Å; and third neighbors 4.5 Å) and by the gap that exists between them which indicates a large amount of crystallinity in the sample. This is clearly a departure from the *ab initio* results where amorphicity was the predominant characteristic after the MD process.

**Figure 12:**
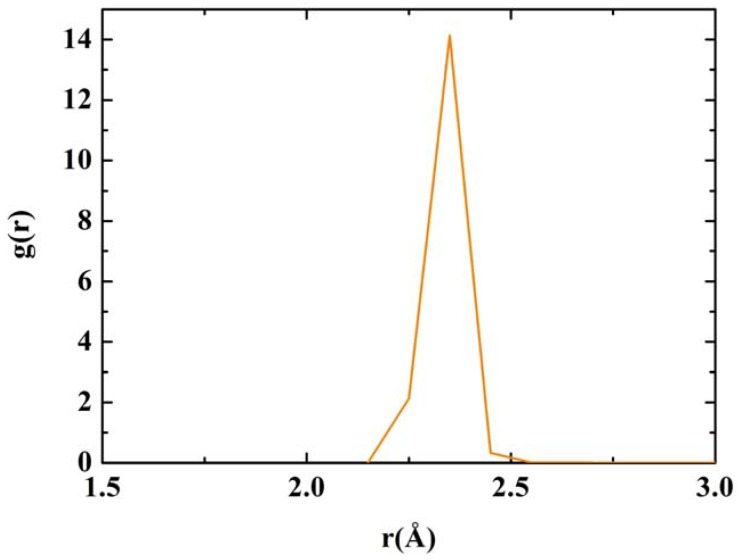
RDF for nanoporous silicon. Fine structure of the first peak of the Tersoff RDF. The prominent peak occurs at 2.35 Å, the crystalline interatomic bond length, a secondary one at 2.25 Å and two minor contributions at 2.45 Å and 2.55 Å.

Assuming the second peak of the Tersoff RDF corresponds to the crystalline second neighbor peak, then the third and fourth crystalline peaks are missing from the Tersoff RDF but we can see a hump around the fifth crystalline peak, close to 6 Å, which is also present in the quantum calculation. Beyond 8 Å (middle-to-long range) the sample behaves like a homogeneous (amorphous) solid. The integral under the first peak of the *J(r)* RDF gives a coordination number of 2.85, much less than for the case of c-Si or a-Si, and suggests the existence of graphitic-like structures for nanoporous silicon. In Figure 12 we have a close up of the first peak of the Tersoff RDF with its main contribution at 2.35 Å, a secondary one at 2.25 Å and two minor contributions at 2.45 Å and at 2.55 Å.

**Figure 13:**
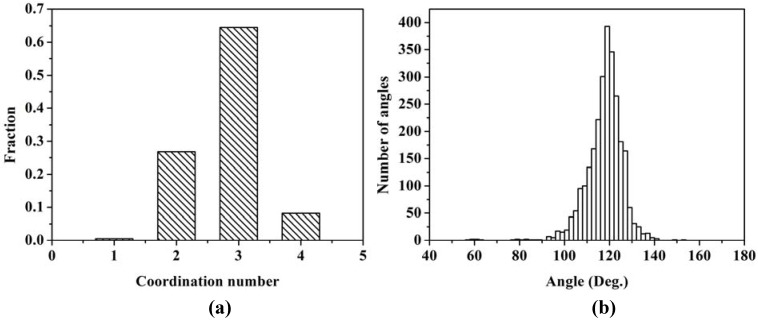
(a) Distribution of twofold, threefold and fourfold coordination numbers. (b) Distribution of bond angles. The first coordination layer is determined by the minimum between the first and the second peak of the RDF, 2.75 Å.

With the bond length defined as the minimum (zero) between the first and the second peaks, 2.75 Å, the next step is to determine the fraction of atoms bonded to 0, 1, 2, 3, *etc*. neighbor atoms. The histogram is shown in Figure 13 (a).

The majority of the atoms are bonded to three nearest neighbors indicating the formation of a graphite-like layered structure of silicon atoms. A significant fraction is bonded to only two, indicating the formation of chains of silicon atoms. Figure 13 (b) shows the distribution of bond angles. The average angle is 117.88°, with a deviation of ±8.00°, a number close to 120°, which supports the existence of atomic layers.

**Figure 14:**
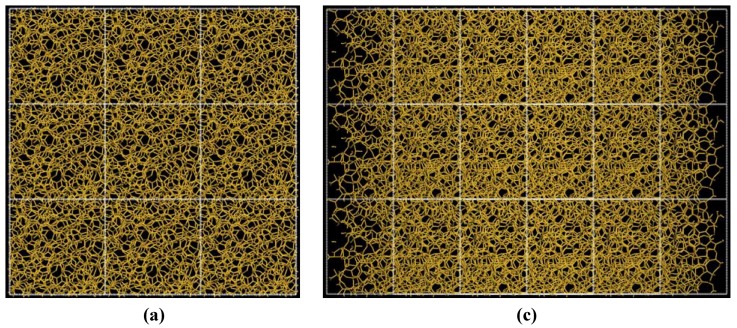

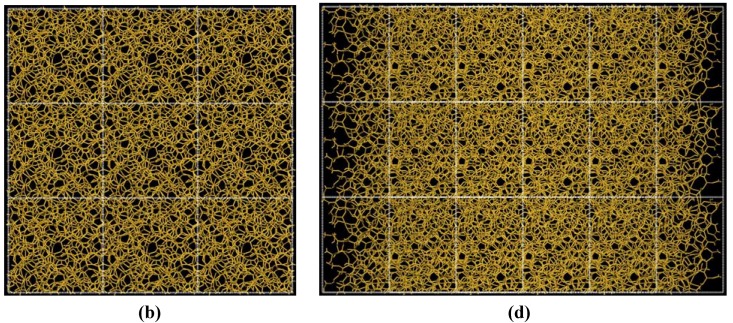
Structure of porous silicon for 3 × 3 × 3 supercells. Small through pores appear along the directions (a) y, (b) z, (c) xz and (d) xy. There may be dendritic pores.

The pores obtained may be dendritic since there does not seem to exist clear through pores in the resulting structure, Figure 14. Small through pores appear along the y, z, xz and xy directions, as shown in Figure 14. These directions are defined with respect to the coordinate system of the supercell vectors.

It is known that the Tersoff potential has several local energy minima that tend to lock the structures at energies that are not necessarily the minimum. This means that applying an MD process to the 1,000 atom supercell at 300 K may be inadequate to reach a general energy minimum, since the atoms do not have enough energy to sample the configuration space available, unlike what occurs with the *ab initio* calculation where the code FASTSTRUCTURE is designed to do precisely this sampling. Clearly more work is needed by increasing the MD constant temperature to see if there is a value that leads to a more adequate minimum energy structure.

#### 3.2.3. Tersoff-Based Vibrational Density of States of Nanoporous Silicon [36]

**Figure 15:**
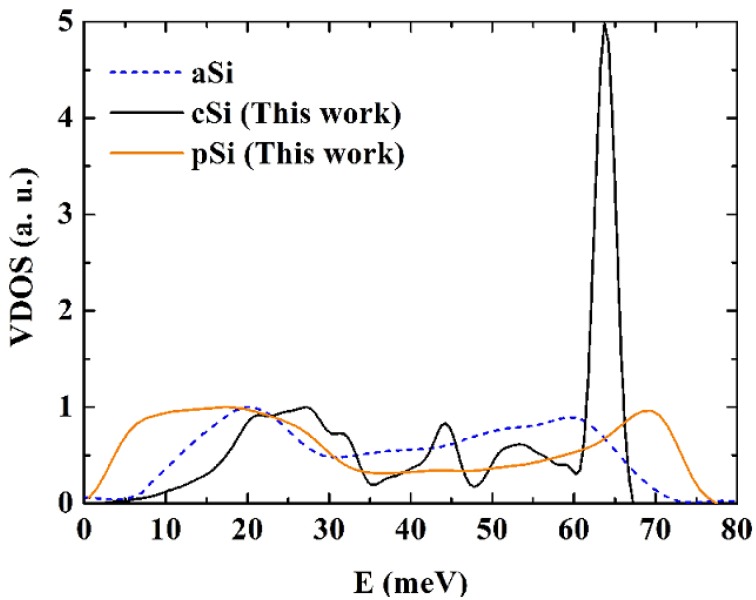
Vibrational densities of states (VDOS) for crystalline, c-Si, amorphous, a-Si [50], and porous, p-Si, silicon calculated using the Tersoff potential. The height of the optical crystalline peak masks the detailed structure of the porous and the amorphous samples. The units on the vertical axis are arbitrary.

**Figure 16:**
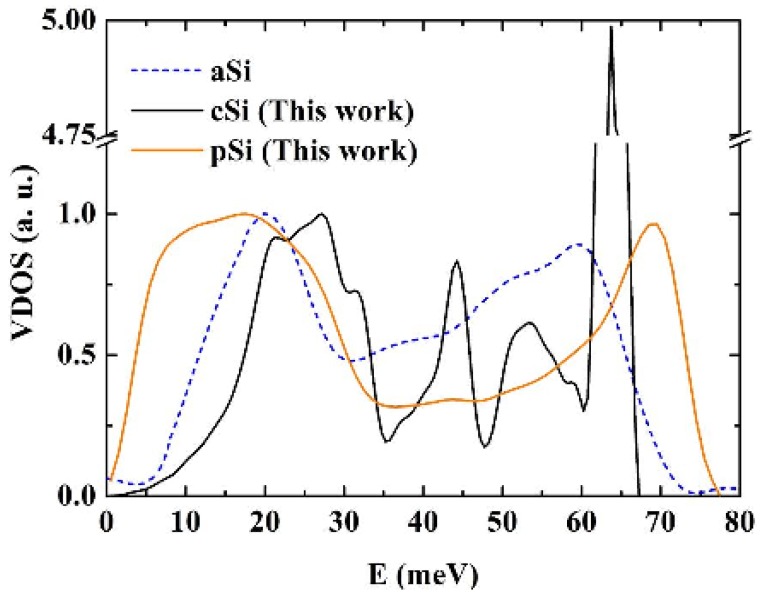
Vibrational densities of states (VDOS) for crystalline, c-Si, amorphous, a-Si [50], and porous, p-Si, silicon calculated using the Tersoff potential. The structures of the porous and the amorphous samples are enhanced. The units on the vertical axis are arbitrary.

For an analysis of the dynamical properties of p-Si, we calculated the vibrational density of states (VDOS) of the generated structure. The vibrational eigenvectors and eigenvalues are found by diagonalizing the dynamical matrix, a 3,000 × 3,000 matrix, within the harmonic approximation. The vibrational frequencies are found using the Monkhorst and Pack [53] scheme, which generates a uniform distribution of q^3^ k-points within the first Brillouin zone. The frequencies are obtained for q = 1, 2, and 3 and converged for q = 1, the Γ point approximation. In Figure 15 and Figure 16 we compare our results for the VDOS of p-Si with our results for the VDOS of c-Si, also using the Tersoff potential and the 1,000 atom crystalline supercell. For the purpose of comparison, recent simulational frequencies of a 216 atoms supercell [50] are also included in the figures. The Tersoff potential has proven useful in the study of vibrational properties of a-Si and since the frequency spectrum helps characterize materials, it is important to obtain the VDOS of the generated nanoporous silicon structure.

All three curves of Figure 15 and Figure 16 are obtained from Tersoff structures. The VDOS function tends to zero when E goes to zero. A similar result is obtained by Ishimaru *et al.* [47] for the case of amorphous silicon using the Tersoff potential. As observed in previous work, the pores introduce not only low energy vibrational modes but also high energy modes so that the interval spanned by the frequencies in the porous sample is wider than in the amorphous or the crystalline samples [37,39,41,42].

For the crystalline sample the intensity of the transverse optical (TO) peak is notorious (Figure 15) and diminishes considerably in the amorphous and in the porous samples. This peak also broadens towards higher energy modes, above 70 meV. Thus, our results are in agreement with experimental results [54,55] in the sense that the TO peak is the most sensitive to structural changes. Lannin *et al*. [55] associate these changes to differences in the width of the distribution of bond angles. For the amorphous and porous samples the longitudinal acoustic (LA) and the longitudinal optical (LO) peaks are not noticeable and in the porous sample an emphatic plateau region appears. The transverse acoustical (TA) peak broadens and is displaced towards low energies indicating the presence of a large number of low frequency modes. The broadening of the frequency span indicates that a larger amount of frequencies can propagate through the porous structure. The displacement towards low energies indicates that the atoms in the structure are freer to move. This could be due to the fact that the atoms are on the average more bonded to three neighbor atoms than any other number, fewer than in the crystalline or amorphous phases.

### 3.3. Hydrogen Adsorption Energetics in Porous Carbon and Silicon

One of the technologically important reasons to study porous materials is to enquire about their applicability to store hydrogen, the fuel of the future. Due to the ecological drive of governments throughout the globe towards getting cleaner and more efficient energy sources hydrogen has received a lot of attention. Porous carbon and porous silicon are likely candidates to do the storage job and consequently the energetics of hydrogen adsorption has to be investigated. In what follows we shall present some estimates concerning the energetics of hydrogen in porous silicon and carbon.

#### 3.3.1. Energetics of Hydrogen Adsorption in Porous Carbon [56]

Storing hydrogen efficiently is considered a potentially useful method for on-board automotive applications. For this reason the storage of molecular hydrogen in carbon nanotubes, nanoporous carbon (activated and carbide-derived carbons), and other carbon nanostructures is being studied intensively [57]. However, the use of hydrogen in large scale applications has been hindered, among other things, by the difficulty and potential danger in the storing process.

Thermodynamic estimations [58] indicate that, in order for reversible adsorption to take place at room temperature, the binding energy of molecular hydrogen to carbon surfaces should be of the order of 300–400 meV/molecule. Since the binding energy of a hydrogen molecule to a graphitic surface is only about 100 meV/molecule [57], molecules have to interact with a larger number of carbon surfaces to multiply this binding energy. That is the case of hydrogen in porous carbon where we expect to find values higher than the 100 meV/molecule calculated in Ref. [57].

Experiment shows that activated carbon with an average pore diameter of 11.75 Å and a pore volume (for pores with radius < 6.5 Å) of 0.75 cm^3^/g is one of the best carbonaceous materials with a storing capacity of 4.5 wt % at 77 K [59]. Simulations that take into account the contribution of quantum effects on the free energy, suggest that the US Department of Energy (DOE) specifications for the year 2010 (6.5% mass ratio and 62 kg/m^3^ volume density; http://www1.eere.energy.gov/ hydrogenandfuelcells/pdfs/freedomcar_targets_explanations.pdf/) can be approached in nanographite platelets (graphene) [60].

In order to study the energetics of hydrogen adsorbed in nanoporous carbon, periodic supercells with 216 atoms and 50% porosity were constructed with the expanded lattice approach, and *ab initio* techniques were applied to generate the nanoporous material and to calculate the energies involved. The hydrogenation was done first by saturating the dangling bonds of the pore surface and relaxing the structure and second by letting hydrogen enter the pore in molecular form and allowing them to relax and distribute accordingly. The total energy of the hydrogenated samples was then calculated. Following this the hydrogenated samples were stripped of the hydrogen atoms and the total energy of the carbon atoms was obtained. Then the original samples were stripped of the carbon atoms and the total energy of the hydrogens was calculated. From these determinations the energetics of the atomic species was deduced. For more details please consult Ref. 56.

**Figure 17:**
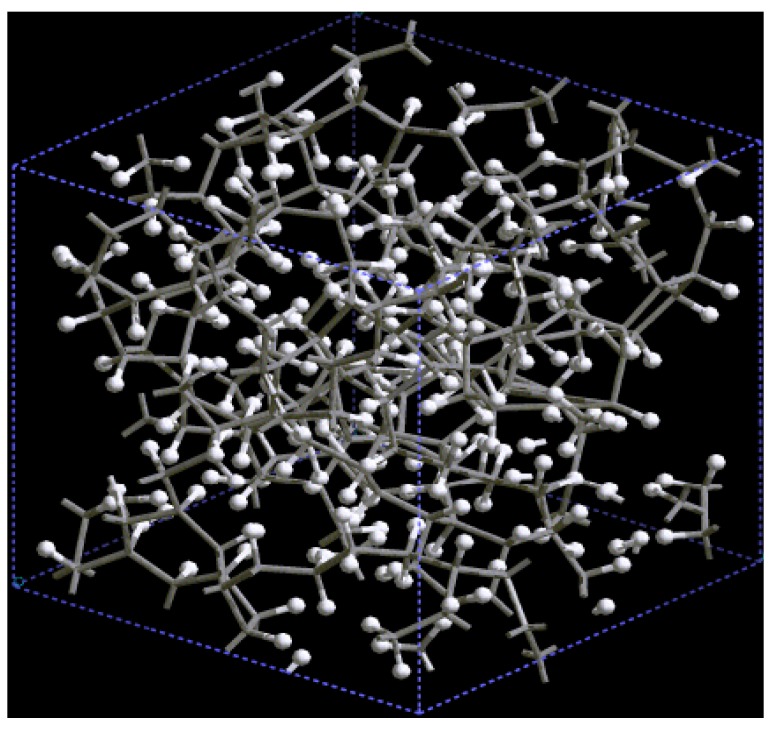
Starting with the relaxed amorphous porous carbon structure, the dangling bonds were saturated with 260 hydrogens and the supercell was geometry optimized.

In Figure 17 the 216 carbon atom supercell whose dangling bonds have been saturated with hydrogens is shown. This saturation required 260 hydrogens and once this was accomplished, the supercell was geometry optimized with **FastStructure** [24]. Evidently, the hydrogens in this process were chemisorbed and using the process described above the result obtained for the chemisorbed hydrogen in porous carbon, for our computationally simulated porous material was 3.16 eV/atom

**Figure 18:**
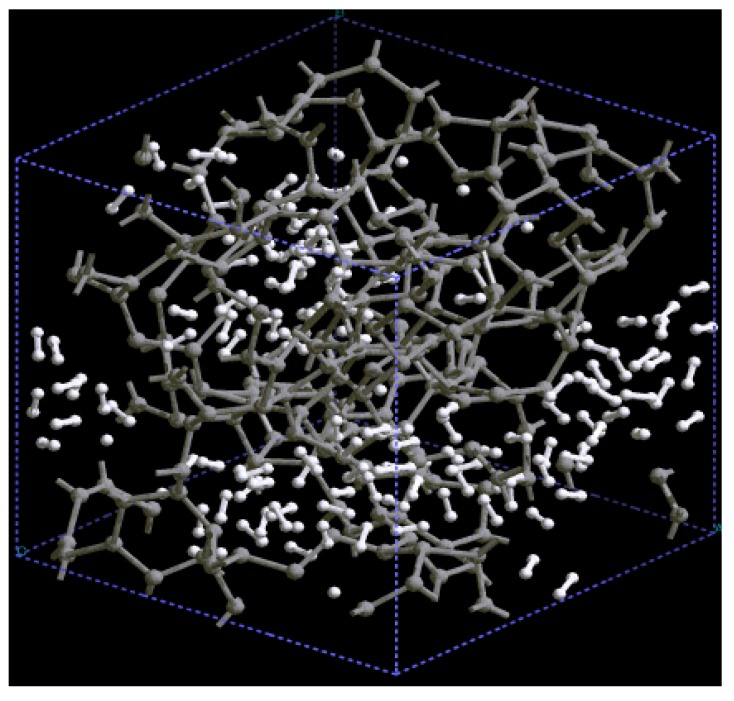
Starting with the relaxed amorphous porous carbon structure, 260/2 hydrogen molecules were constructed within the pore and the supercell was geometry optimized.

To generate a porous structure with hydrogen molecules (Figure 18) we started with the relaxed porous carbon structure, constructed 130 molecules within the cell to yield the same total number of hydrogen atoms, 260, and then relaxed the cell with **Discover** [61]. To calculate the average energy of the CH bond (The default bond length for CH used in the figures is given by the relation 1.15(C _covalent radius_ + H _covalent radius_), or 1.31 Å), we proceeded as for the chemisorbed sample, and the energy obtained was 343.89 meV per hydrogen atom, which compares favorably with values reported in the literature, 300–400 meV/molecule. If we assume that all atoms are in molecular form, then the energy per molecule would be 687.78 meV, much lower that the average energy for chemisorbed hydrogens (3.16 eV/atom).

Much discussion exists concerning the way hydrogen is adsorbed in carbon. For the purposes of storing hydrogen, physisorption seems to be the adequate process since chemisorption implies higher binding energies and therefore larger energies to liberate the hydrogen. Nevertheless although physisorption seems to be the preferred adsorption process, pore-size dependence may alter the incorporation of hydrogen in porous carbon affecting the efficiency of the process.

#### 3.3.2. Energetics of Hydrogen Adsorption in Porous Silicon [62]

Porous silicon may be an interesting alternative to store hydrogen. Unlike carbon, its bonding multiplicity is limited, and because of this, the probability of having more dangling bonds on the pore surface is larger than in carbon, which would increase the number of chemisorbed hydrogens. Porous silicon has been investigated mainly due to its luminescent properties and its potential applicability in optoelectronic devices; little consideration has been given to its potential use as a fuel tank for hydrogen.

Hydrogen may be physisorbed and this would have the advantage of making it more readily available by heating the silicon samples, but also more unstable. Hydrogen may also be chemisorbed at energies of the order of the bonding energy of SiH, 3.29 eV [30], which is 23% smaller than the bonding energy of CH, 4.26 eV [30]. This would make the chemisorption process in porous Si an interesting candidate for making the adsorption of hydrogen more stable than the physisorption in carbon, and also more readily freed than chemisorbed hydrogen in carbon.

In our previous work [2,3] we found that the energies per hydrogen atom in porous carbon were 2.74 eV for the sample with diamond-like density (1.75 g/cm^3^) and 3.10 eV for the sample with graphite-like density (1.31 g/cm^3^). These values are smaller than 4.26 eV, the normal CH bond energy, and it may be expected that the energies per hydrogen atom in porous silicon may still be lower than the textbook value for SiH, 3.29 eV, thereby making chemisorption of hydrogen in this material an attractive option. It appears then that although physisorption seems to be the preferred adsorption process in carbon, it is feasible that chemisorption in porous silicon may become a competitor, with some advantages, like the stability of the hydrogen gas in the material.

**Figure 19:**
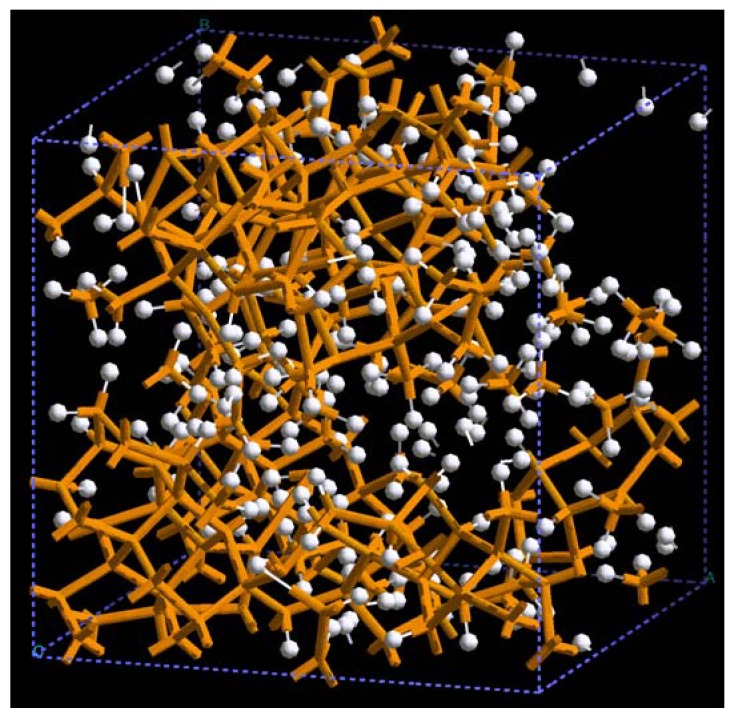
Starting with the relaxed amorphous porous silicon structure, the dangling bonds were saturated with 252 hydrogens. Next the supercell was geometry optimized.

Using nanoporous silicon periodic supercells with 216 atoms and 50% porosity, constructed using the *ab initio* expanded lattice approach [23] and a 10 fs time step, the dangling bonds of the silicon atoms were first saturated with hydrogen. This required 252 hydrogens and subsequently the supercell was geometry optimized, Figure 19. Evidently, the hydrogens in this process were chemisorbed and in order to obtain the average energy per atom we first calculated the total energy of the structure; then we stripped off the hydrogens and the total energy of the remaining silicons was obtained. Next the original sample was stripped of the silicon atoms and the total energy of the hydrogens calculated. The result obtained for the average energy of hydrogen chemisorbed in porous silicon is 2.50 eV/atom, to be compared with 3.16 eV/atom for hydrogen chemisorbed in carbon.

**Figure 20:**
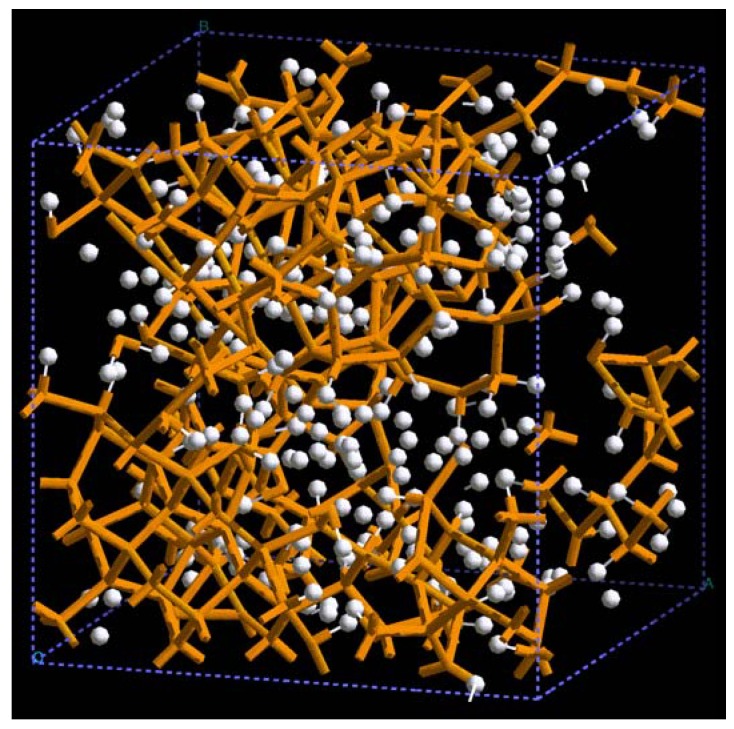
Starting with the relaxed porous Si structure, the dangling bonds were saturated with hydrogens displaced 1 Bohr radius along the (1, 1, 1) direction. Next the supercell was geometry optimized.

To generate a porous structure with hydrogens inside the pore (Figure 20) we started with the relaxed silicon structure, attached hydrogens to fill the dangling bonds and then displaced them one Bohr radius along the (1,1,1) direction. An MD process of 100 steps at 300 K, with the previous time step, was then applied to this sample and finally the geometry was relaxed. To calculate the average energy of the hydrogens we proceeded as before, and the energy obtained was 1.66 eV/atom, lower that the average energy for chemisorbed hydrogens

## 4. Metallic Elements

The application of the expanded lattice approach to covalent semiconductors like silicon and carbon demonstrates that since the pores are formed due to energetic considerations during the MD process at constant temperature, they would appear along the directions that satisfy these energetic considerations and one can study the preferentiality of the orientation in the pore forming process. When porous silicon is formed by carving a sample of crystalline silicon the orientation of the pore is predetermined and the preferentiality cannot be easily studied.

Furthermore, the fact that the expanded low density cell dynamically evolves due to the forces that act within the system, one expects that the final structure would not be closer to the crystalline state except for densities that are comparable to a certain crystal structure. For example, for densities close to the graphite density the carbon atoms may show some degree of crystallinity even if it is far from the density of diamond. Therefore, in general, an amorphous structure is to be expected when the densities are notably below those corresponding to the crystalline state, and that is what we observe.

So the question arises: what would happen if we apply the expanded lattice approach to metallic systems? Unlike semiconducting systems, metallic materials are not bound by covalent bonds and since the metallic bond is very different from the covalent one, it would not be obvious that the expanding lattice approach would work for metal systems. Since porous metallic systems are very important from the technological viewpoint it occurred to us that we should investigate the lattice expansion approach as applied to metals and alloys to better understand their structure and properties. In what follows, we present some recent results of the application of our expanding approach to aluminum and gold with supercells of 108 atoms.

### 4.1. Nanoporous Aluminum

**Figure 21:**
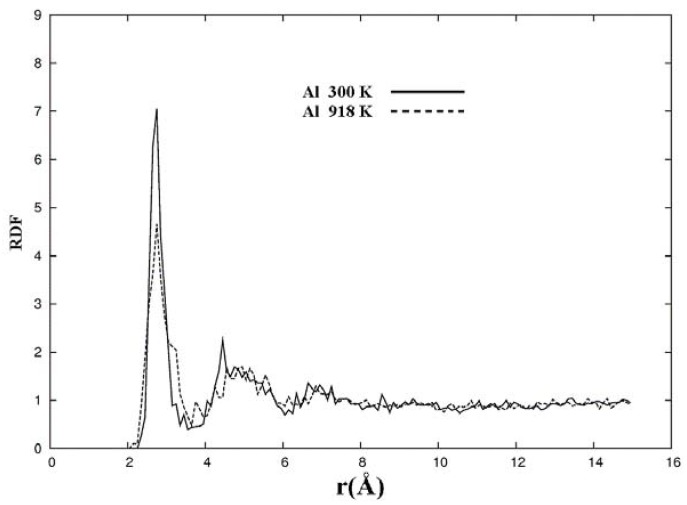
Radial (pair) distribution functions, RDFs, for 50% porosity supercells with 108 aluminum atoms after 500 steps of MD processes at 300 K and at 918 K. See text.

Aluminum is a very useful metal in industry and we have studied it in pure amorphous form [63] and alloyed with silicon [64] and nitrogen [65]. So it is only natural to consider the porous form obtained by expanding the lattice and applying an MD process to the 108-atom expanded crystalline fcc lattice. Its melting temperature is 933 K at atmospheric pressure so we did MD at 918 K and at 300 K. The parameters used were a dnd basis set, no pseudopotential with the Harris functional, the VWN local density approximation, and a 7 fs time step. The cutoff radius for the wave functions was 5.00 Å and an extra fine numerical integration grid was utilized. Since random velocities were used to guarantee a stochastic process it would seem likely that the final structures would not importantly depend on the original distribution of velocities. In Figure 21 the RDFs of the porous structures generated at 300 K and at 918 K are shown. It can be seen that for the structure generated at 918 K the second and third peaks of the RDF are relatively less pronounced than for the corresponding RDF of the structure generated at 300 K. Between about 10 and 13 Å the values of the RDFs go below 1 signaling the presence of voids, a feature that we have found in other porous materials. The behavior of the second and third peaks of the 918 K RDF seems to indicate that the amorphous structure found for 300 K becomes a more homogeneous, liquid-like structure at 918 K. However the decrement below the value of 1 indicates the presence of pores that would be difficult to understand in a liquid-like structure. We believe this behavior is due to the presence of lamellae structures observed in this sample (Figure 23), which may be precursors to the formation of foams in aluminum, but more studies are needed to understand these results.

**Figure 22:**
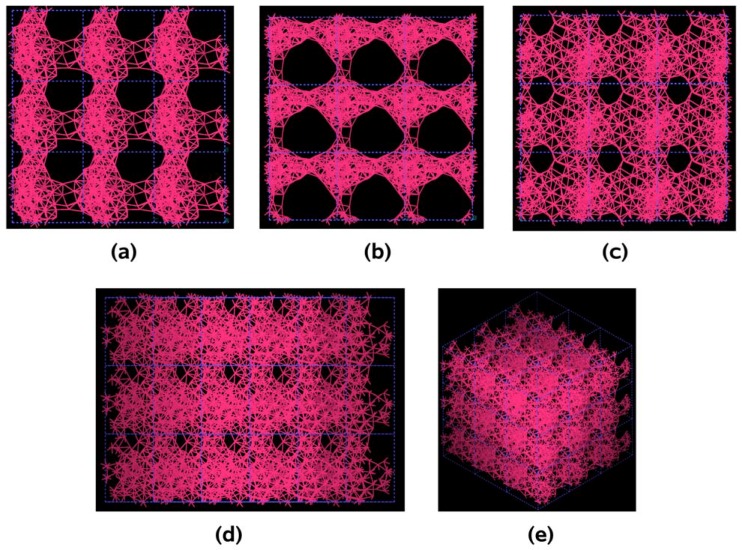
Pores formed in a 3 × 3 × 3 50% porosity aluminum supercell when it undergoes an MD process at 300 K during 500 steps. There are prominent through pores along (a) x, and (b) y directions, and smaller pores along (c) z, (d) xz and (e) xyz directions.

**Figure 23:**
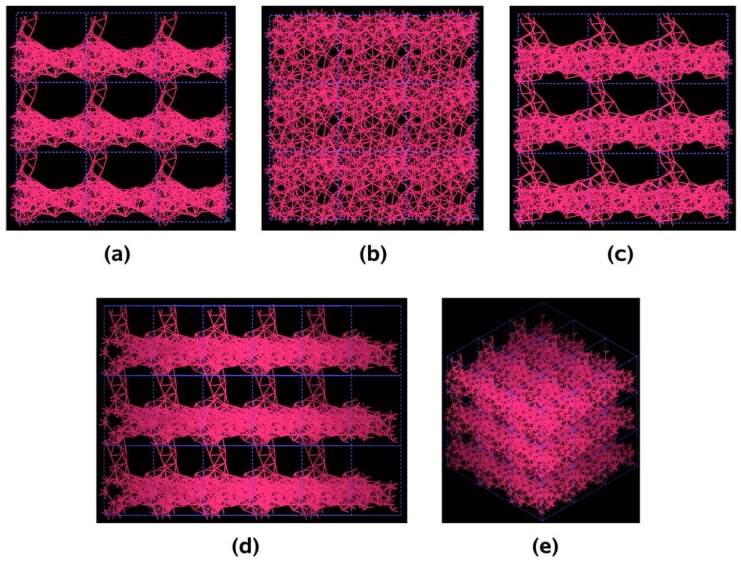
Pores (layers) formed in a 3 × 3 × 3 50% porosity supercell with 108 aluminum atoms when it undergoes an MD process at 918 K during 500 steps. There are prominent through pores, almost layers, along (a) x, (c) z and (d) xz directions.

In Figure 22 and Figure 23 the final structures of the nanoporous aluminum samples obtained at 300 K and at 918 K are shown. To illustrate the presence of pores in the systems studied, we chose to represent a larger porous cell by 3 × 3 × 3 supercells along the three spatial directions, as for semiconductors; this may give the idea that we have a periodic material but this periodicity is a consequence of the supercell approach and the amorphous/porous structure is contained within a given supercell. The structures are almost layered, perhaps precursors of aluminum foams, and can be readily observed; due to the limited number of atoms (a rather small supercell) the size of the “pores” are in the nanoregime. Here again there are no external influences on the direction of the pores formed and what we see is what energetically would occur in samples that undergo *ab initio* MD at those two temperatures.

### 4.2. Nanoporous Gold

Nanogold has properties that are astonishingly different from bulk gold. Reactivity for one is present in the nanoregime and this makes it very interesting from the biomedical viewpoint in particular, and from the technological viewpoint in general. So it is important to consider the porous form obtained by expanding the lattice and applying an MD process to the 108 atom expanded crystalline fcc lattice. Its melting temperature is 1,337 K at atmospheric pressure and we did MD at 1,320 K and at 300 K. The parameters used are a dnd basis set, dspp pseudopotential with the Harris functional and the VWN local density approximation; a 10 fs time step was also used. The cutoff radius for the wave functions is 5.00 Å and an extra fine numerical integration grid was utilized. Since random velocities were used to guarantee a stochastic process it would seem likely that the final structures would depend only to a certain extent on the original distribution of velocities.

**Figure 24:**
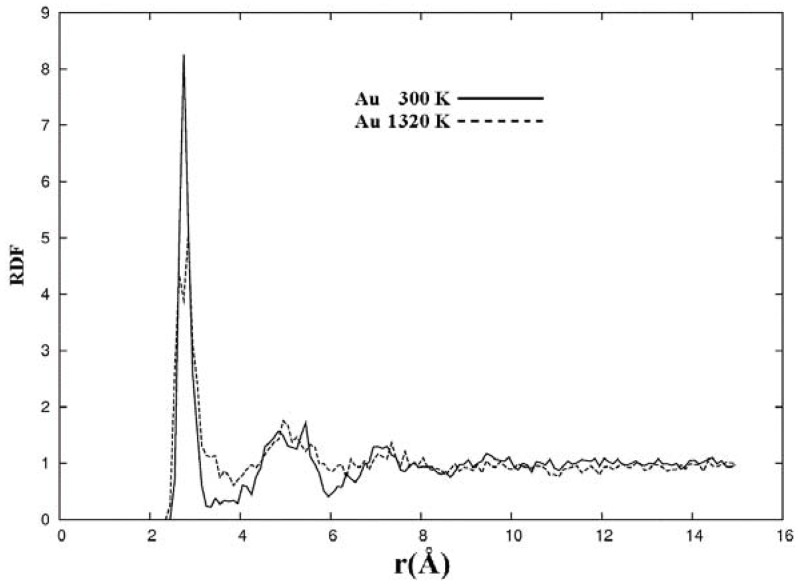
Radial (pair) distribution functions, RDFs, for 50% porosity supercells with 108 gold atoms after 500 steps of MD processes at 300 K and at 1,320 K. See text.

In Figure 24 the RDFs of the porous structures generated at 300 K and at 1,320 K are shown. Similar to what occurs in aluminum the structure generated at 1,320 K has relatively less pronounced second and third peaks compared to the corresponding RDF of the structure generated at 300 K. Between about 10 and 14 Å the values of the RDFs go slightly below 1 signaling the presence of voids for gold, not as prominent as for aluminum. Here also, the behavior of the second and third peaks of the 1,320 K RDF seems to indicate that the amorphous structure found for 300 K becomes a more homogeneous, liquid-like structure; however the slight decrement below the value of 1 indicates the presence of pores that would be difficult to understand in a liquid-like structure. More studies are needed to comprehend these results.

**Figure 25:**
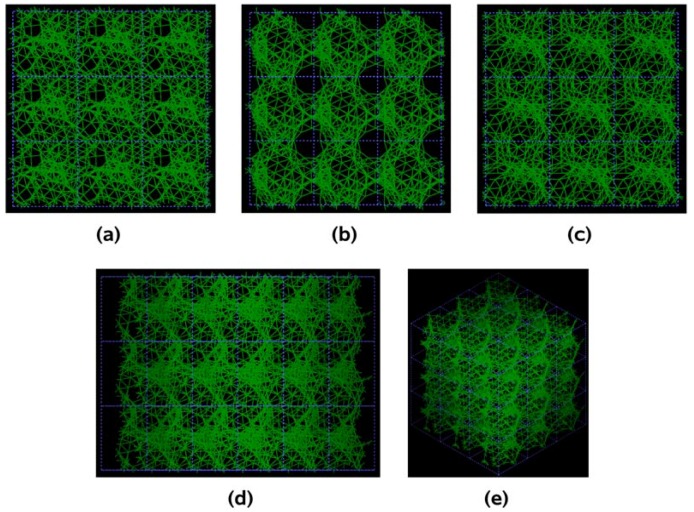
Pores formed in a 3 × 3 × 3 50% porosity, 108-gold atom supercell when it undergoes an MD process at 300 K during 500 steps. There are through pores along the (b) y direction, and a tendency to through pore formation along all the other directions.

**Figure 26:**
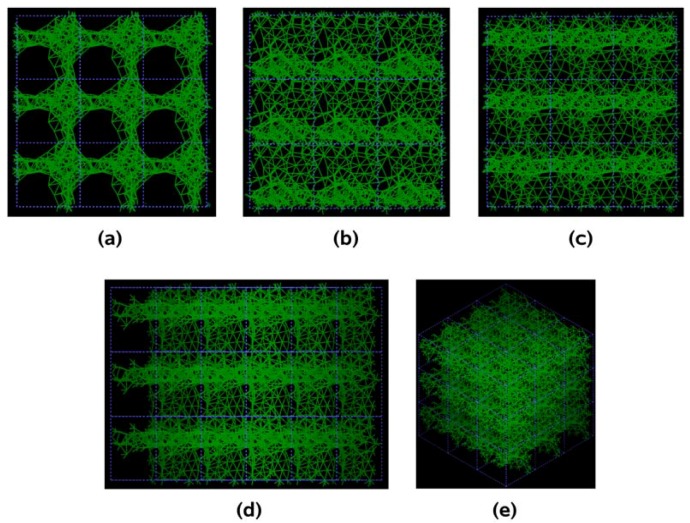
Pores formed in a 3 × 3 × 3 50% porosity, 108-gold atom supercell at the end of 500 MD steps at 1,320 K. Prominent through pores appear along the (a) x direction, and a tendency to through pore formation along all the other directions.

In Figure 25 and Figure 26 the final structures of the nanoporous gold samples obtained at 300 K and at 1,320 K are shown. The periodicity observed is a consequence of presenting a cell of 3 × 3 × 3 supercells and the amorphous/porous structures are contained within each supercell of 108 atoms. Some pores can be observed but there are not as many as for the aluminum case. However more careful revisions of Figure 25 and Figure 26 indicate the existence of voids (not necessarily through pores) in the material and perhaps dendritic pores. Due to the limited number of atoms (a rather small supercell) the size of the pores are in the nanoregime. Here again there are no external influences as to the direction of the pores formed and what we see is what energetically would occur in samples that undergo *ab initio* MD at those two temperatures.

## 5. Conclusions

The expanding lattice method presented here provides computationally generated nanoporous structures that lay somewhere between the crystalline porous materials and the amorphous ones. This may be due to the porosity considered (50%) or to the small number of atoms in the supercell (a few hundreds). One would expect that the larger the number of atoms for a given porosity, the larger the backbone for a large pore material, and this would lead to a structure more crystalline than amorphous. However, if the given porosity is caused by a large number of small pores the backbone would be less determining in the structure, and amorphicity may set in.

The *ab initio* carbon samples have structures in which single, double and triple bonds are important whereas in the *ab initio* silicon samples this multiplicity does not occur due to the non-versatility of the silicon bond. The most probable interatomic distance, *i. e.*, the position of the maximum of the first peak for nanoporous carbon, is smaller than that of the diamond structure, while the most probable interatomic distance for silicon is larger than its crystalline counterpart. This implies a contraction for nanoporous carbon and an expansion for nanoporous silicon with respect to their crystalline counterparts. Pores in the *ab initio* silicon structures appear mainly along the x, y, z and yz and to a lesser extent along the xyz directions. For carbon the corresponding *ab initio* pore structure is less clear but tubular through pores are observed in the x and z directions and smaller pores along the y, zy and xyz directions. For lower density carbon (61.1% porosity nanoporous carbon with a density of 1.38 g/cm^3^) some pores exist along z, yz and xyz and smaller ones along x and y directions. It would seem that porous carbon tries to avoid the voids (pores) by expanding and filling as much as possible the available space whereas for silicon the opposite seems to occur.

An interesting phenomenon appears when we generate porous structures using the Tersoff potential. This classical approach on a 1,000-atom periodic supercell at 300 K leads to nanoporous structures apparently with less through pores than the *ab initio* ones and with well localized first and second neighbors indicating a tendency to crystalline-like structures in the short-to-middle range. The position of the nearest neighbors’ peak coincides with the crystalline value, 2.35 Å, whereas the position of the second peak is larger than the corresponding second neighbor peak in crystals: 4.05 Å *versus* 3.84 Å, respectively. At distances greater than 8 Å (middle-to-long range) the sample is homogeneous (non-crystalline). Since the Tersoff potential contains several parameters it may have relative minima corresponding to each parameter and the low energy structures that appear may be due to the locking in of the final structure in one of these wells and therefore give the wrong idea about the “lowest energy structure” reached. Studies are under way to see if, in fact, the structures here presented are the lowest possible energy structures compatible with the overall conditions.

The use of the Tersoff potential to calculate the vibrational density of states shows that the tendency in going from crystalline to amorphous is maintained in going from amorphous to porous; in other words, as the density diminishes the span of the VDOS increases and noticeably more modes are found at low frequencies than at high frequencies. The tendency to develop a middle-of-the-range plateau in the amorphous is highly enhanced in the porous. Tersoff predicts the formation of layers and chains of silicon atoms but this may be due to the fact that the structure is not a minimum energy structure as mentioned above. It is clear that more calculations are necessary, varying temperature and porosity, to discern under what circumstances the Tersoff potential is apt for describing the static and dynamic structures of nanoporous silicon.

The results obtained from the energy calculations indicate that the average energy of hydrogen in silicon is smaller than the average energy of hydrogen in carbon. Two samples of hydrogenated porous carbon, constructed in two different ways have been analyzed. In the first sample the energy found for chemisorbed hydrogen is of the order of the energy of the normal CH bond and higher that the corresponding energy for hydrogen chemisorbed in silicon. For the second sample we found an energy value of 343.89 meV per hydrogen atom which is in good agreement with those reported in the literature [57]. Two samples of hydrogenated porous silicon constructed in two different ways have also been analyzed to investigate whether, under the particular conditions considered, the predominant adsorption process for hydrogen in silicon is physisorption or chemisorption. We find that the average bonding energy SiH obtained is below the typical value reported for SiH bonds in molecules and compounds. The result for the average energy of hydrogen chemisorbed in porous silicon was 2.50 eV/atom, to be compared with 3.16 eV/atom for hydrogen chemisorbed in carbon.

The metallic elements evolve into interesting atomic structures. For aluminum at 300 K the final structure displays prominent pores along the x and y directions, and smaller pores along the z, xz and xyz directions. When the MD process is done at 918 K layered structures appear (that could be precursors of the foamy structures found for this element) with very large pores located along the directions x, z and xz directions. When the RDFs for this material at the two different MD temperatures are analyzed, a tendency to the formation of a more homogeneous atomic structure is observed manifested by the lesser preponderance of the second and third peaks of the 918 K RDF compared with the corresponding RDF of the structure generated at 300, while exhibiting a porous structure. This certainly does not correspond to some sort of a melt but to layered structures that could be precursors to the formation of foams for this material; work is in progress to investigate this feature. Finally, for gold when the MD is done at 300 K there are through pores along the y direction and a tendency to through pore (or layer) formation along the other 4 directions. When the MD is carried out at 1,320 K prominent through pores appear along the x direction and a tendency to through pore or layer formation (dendritic pores?) appears along the other 4 directions. As for aluminum, the structure generated at 1,320 K has relatively less pronounced second and third peaks compared to the corresponding RDF of the structure generated at 300 K, however voids do seem to exist, witnessed by the behavior of the RDFs between 10 and 14 Å. Clearly these results have to be confronted with other studies that give complementary information in order to fully characterize the structures.

The advantage of our approach is that we can generate “freely” (minimum energy restricted) forming nanoporous structures of semiconducting and metallic materials in contrast to the generation of structures by reverse processes that fit some specific experimental results, or to the generation of structures by incorporating *ad hoc* structural elements that presumably should appear in the real material.
